# Tablet-based versus presentation-based seminars in radiology: Effects of student digital affinity and teacher charisma on didactic quality

**DOI:** 10.3205/zma001641

**Published:** 2023-09-15

**Authors:** Sandra Weigel, Joy Backhaus, Jan-Peter Grunz, Andreas Steven Kunz, Thorsten Alexander Bley, Sarah König

**Affiliations:** 1 University Hospital Würzburg, Institute for Medical Teaching and Medical Educational Research, Würzburg, Germany; 2 University Hospital Würzburg, Department of Diagnostic and Interventional Radiology, Würzburg, Germany

**Keywords:** teaching, radiology, tablets, educational technology, medical education

## Abstract

**Aims:**

Tablets are being adopted as teaching medium in medical education more frequently. Here we compared two teaching formats in a radiology seminar using a tablet-based student-centred approach guided by teachers and traditional presentation-based, teacher-centred instruction. The aim was to investigate the effects on academic performance, estimated learning gain, didactic quality, as well as how teacher charisma and student digital affinity influence these elements.

**Methods:**

Data from 366 students were collected. Student digital affinity, didactic quality of, and overall satisfaction with the seminars were rated for each teaching format over three semesters, whereby in the last semester, students additionally estimated their learning gain, took a knowledge and image interpretation test, and rated teacher charisma.

**Results:**

The tablet-based seminars yielded significantly higher ratings for didactic quality and overall satisfaction. However, the presentation-based seminars proved superior with respect to academic performance as well as estimated learning gain. When employing tablets, teacher charisma correlated with estimated learning gain, and digital affinity affected didactic quality. Additionally, good seminar organization, comprehensible learning objectives, and optimal variation of learning activities were identified as important factors.

**Conclusion:**

This study suggests a complex interplay of various factors concerning teachers, students, and didactics that can be assessed and improved to assure the successful curricular implementation of tablets. Of note, tablet integration and thereby active engagement of students with imaging analysis skills does not automatically result in greater declarative knowledge. Nevertheless, understanding the complexities of structuring and delivering tablet-based, teacher-guided instruction is essential to creating meaningful educational experiences.

## Zusammenfassung

**Zielsetzung:**

Tablets werden zunehmend als Lehrmedium in der medizinischen Ausbildung eingesetzt. Hier vergleichen wir zwei Lehrformate im Radiologie-Seminar: ein Tablet-basiertes, Studierenden-zentriertes Format, das von Dozierenden begleitet wird, und ein traditionelles Präsentations-basiertes, Dozierenden-zentriertes Format. Ziel war es, die Auswirkungen auf den objektiven Lernzuwachs, den selbsteingeschätzten Lernzuwachs und die didaktische Qualität sowie den Einfluss des Charismas der Dozierenden und der Technikaffinität der Studierenden auf diese Elemente zu untersuchen.

**Methodik:**

Daten von 366 Studierenden wurden erhoben. Die Technikaffinität der Studierenden, die didaktische Qualität und die allgemeine Zufriedenheit mit den Seminaren wurden für jedes Lehrformat über drei Semester bewertet. Im letzten Semester schätzten die Studierenden zusätzlich ihren Lernzuwachs ein, absolvierten ein Wissens- und Bildinterpretationstestat und bewerteten das Charisma der Dozierenden.

**Ergebnisse:**

Die Tablet-basierten Seminare erzielten signifikant bessere Bewertungen für didaktische Qualität und allgemeine Zufriedenheit. Die Präsentations-basierten Seminare schnitten jedoch in Bezug auf den objektiven und selbsteingeschätzten Lernzuwachs besser ab. Bei Verwendung von Tablets korrelierte das Charisma der Dozierenden mit dem selbsteingeschätzten Lernzuwachs, und die Technikaffinität beeinflusste die didaktische Qualität. Darüber hinaus wurden gute Seminarorganisation, verständliche Lernziele und optimale Variation der Lernaktivitäten als bedeutsame Faktoren identifiziert.

**Schlussfolgerung:**

Diese Studie legt ein komplexes Zusammenspiel verschiedener Faktoren aus Dozierenden, Studierenden und Didaktik nahe. Es sollte beachtet und ggf. moduliert werden, um eine erfolgreiche curriculare Implementierung von Tablets zu ermöglichen. Jedoch zeigte sich, dass die Integration von Tablets und damit das aktive Einbinden der Studierenden in die Bildanalyse nicht automatisch zu mehr deklarativem Wissenserwerb führt. Das Verständnis der komplexen Strukturierung bei der Umsetzung von Tablet-basiertem, von Dozierenden geleitetem Unterricht ist grundlegend, um optimale Lernbedingungen zu schaffen.

## 1. Introduction

With the rise of new media at the beginning of the new millennium, the use of portable tablets in particular seemed promising in clinical routine [1]. Various studies have since documented numerous positive effects of their use in clinical settings from both the physicians’ as well as the patients’ point of view. Tablets were shown to improve information access, enhance workflow, simplify documentation, and increase physicians’ time for patient care [2], [3], [4]. Teaching with tablets has also attracted attention as an effective tool in medical education. Studies indicate that digital devices can promote learner-centred education by facilitating peer feedback, knowledge sharing and discussion [5], positively affect attendance [6], and induce high levels of student satisfaction [7]. Such novel teaching approaches may be of particular interest in the field of radiology, since medical students view the lack of clear and structured radiological evaluation of imaging and interpretation of findings as one of the most common shortcomings in their medical studies [8], [9]. We postulate that an essential difference between theoretical radiological education (factual knowledge) and the practical implementation of radiological image interpretation in a clinical context exists, necessitating students to navigate through patient datasets (application of knowledge). When integrating tablets in radiological teaching, students can learn to operate a picture archiving and communication system, thereby increasing the degree of task-authenticity. Students gain practical learning experiences that mimic and reflect the characteristics, complexity, and demands of future workspace tasks in imaging. Nonetheless, a dearth of evidence remains regarding tablet-based teaching, despite some studies pointing towards its potentially positive impact on learning outcomes [10], [11], while others find no difference compared to radiology courses taught conventionally [12], [13].

Structured delivery of knowledge and skills, the use of appropriate teaching and learning methods as well as media in the classroom, and active involvement of students are facets that are widely used as surrogate parameters for didactic quality [14]. Yet there is still a lack of information on the role of teacher charisma in education. We postulate that there is a positive influence on inducing student learning interest [15], engagement [16], and learning effectiveness [17], [18]. A link to a desirable outcome such as critical thinking, cognitive development, and student satisfaction was also recognised [19], [20]. A recent study into teacher perceptions of digital technologies documented that using technology in teaching may also help with motivating students [21]. However, the mechanisms that underpin the role of teachers in technology-based education are not yet fully understood. A study conducted in a computer-based learning environment documented that charismatic trainers influence skills acquisition by boosting positive affectivity [22]. Albeit pushing the teacher into a more moderating and prompting role in tablet-based seminars, we predicted that teacher charisma would continue to have a significant impact on learning outcomes.

A misalignment between the expectation of students being innate, talented users of digital technologies and the often misjudged actual levels of digital competence experienced in classroom settings may act as an obstacle in digital teaching formats. Studies therefore suggest that a more sophisticated review of students’ digital affinity is necessary to implement digital devices in the most effective way to support learning [23], [24], [25]. A recent study approached this challenge by identifying two clusters of students differing in digital affinity and found an interaction effect between the digital affinity cluster and the learning outcome in different formats of knowledge delivery [26]. However, a gap in understanding remains as to how digital affinity may affect students’ evaluation of teaching and course quality when digital devices and media are employed in classroom activities.

The aim of this study was to compare and contrast two different teaching formats in radiology seminars using tablet-based student-centred and teacher-guided instruction versus presentation-based and teacher-centred instruction. In order to explain the impact of tablets on teaching and learning, this study seeks to obtain data, which will help to address the following questions:

- How do the different teaching formats compare in estimated learning gain, academic performance, perceived teacher charisma, didactic quality, and student satisfaction?
- How does the teaching format affect the influence of teacher charisma and student digital affinity on the other variables, especially on didactic quality?

## 2. Materials and methods

### 2.1. Study design and participants

This prospective cross-sectional study was conducted at a medical school in Germany offering a standard six-year curriculum comprising two preclinical years of teaching, three clinical years, and one practical year of training. In the fourth year, teaching in radiology comprises lecture series and twelve obligatory seminars, of which six were included in this study: Gynaecological and paediatric imaging were both taught in a presentation-based format using visuals produced in Microsoft PowerPoint (Redmond, USA). In contrast, the four seminars on imaging of the abdomen, heart and large vessels, thorax, and musculoskeletal system were presented in a tablet-based format. Students used anonymised patient imaging sets via a specialized picture archiving and communication system (PACS) application (Merlin, Phoenix-PACS, Freiburg im Breisgau, Germany). Twelve to fourteen different students each week attended the seminars of 90 minutes’ duration and were taught by 17 radiology residents from the local department of radiology. Each of the six seminars was taught by various residents, and the majority of the residents (58.8%) taught seminars on at least two different topics. Thanks to a rotation schedule, the seminars ran in a different order for each student group, guaranteeing each student's participation in both the presentation-based and the tablet-based seminars. Participation in the study was on a voluntary basis with no disadvantage resulting from not participating.

### 2.2. Teaching methods

All seminars were based on the same didactic concept: basic knowledge in anatomy and radiology relevant to the topic were recapitulated and followed by presentations of the real patient cases, each comprising a medical history, the clinical question investigated, and the radiological imaging data sets (radiography and computed tomography). The two formats of the seminars were labelled to outline the medium that teachers used for instruction: presentation and tablet. In the traditional presentation-based seminars, the teacher embedded the images in the visuals and actively involved the students by jointly interpreting the images, trying to involve as many students as possible. The tablet-based seminars were considered as student-centred and teacher-guided instruction. In addition to small introductory prompts given by the lecturer, it was organised as partnered sessions: two students shared a device, allowing them to perform hands-on tasks of imaging analysis such as selecting appropriate image reconstructions, zooming, and scrolling. After evaluating the data, the image interpretation and the correct diagnosis were discussed in the plenary session. Up to 17 patient cases were prepared for each seminar; owing to time restrictions, only a selection could be used.

### 2.3. Measures and procedure

The study ran during three semesters (winter term of 2016/17, summer term of 2019, and winter term of 2019/20). In all semesters, students were asked to participate in an online end-of-semester survey on the platform EvaSys (Lüneburg, Germany) to rate their personal digital affinity, didactic quality of, and overall satisfaction with the seminars. Only in the last semester (winter term of 2019/20), students additionally estimated their learning gain, rated teacher charisma, and took a knowledge and image interpretation test by means of a smartphone-based quick-response questionnaire at the end of each session. Data from the end-of-semester and quick-response surveys were matched using the matriculation numbers, which were provided by the students. Table 1 (Tab. 1) lists all the variables used in this study, their definitions, methods of measurement, scale values, and value labels.

The estimated learning gain of each seminar was assessed before the students completed the knowledge and image interpretation test. The gain was calculated based on four operationalised learning objectives (see attachment 1 ). Students rated their level of competence in each learning objective on a six-point Likert scale from “strongly agree” to “strongly disagree” using a retrospective post-then-pre design (on completing the seminar, students rated their competence both after and before the seminar). The mean competence level was expressed as a percentage. Thus, the difference was taken to calculate the estimated learning gain using a weighted gain method, which was defined as the percentage raw difference multiplied by a weighting coefficient to adjust for pre-test variability [27].

To measure academic performance, tests assessed the clinical knowledge and imaging interpretation skills of the students. Thus, 24 questions were selected from former curricular examinations, which were identified as best meeting good criteria of item difficulty ranging from 0.4-0.8 and corrected item-total correlation exceeding 0.3, then revised and matched to the topics of the seminars. Each seminar test comprised four to five questions; responses were set as 5-option single-choice or multiple-true-false. Students received one point for the correct answer and half a point for selecting more than 50% of the correct responses in the multiple-true-false-type questions. The total test score was expressed as a percentage. In order to prevent teaching to pass the test, the teachers were not aware of the test questions.

To design the study questionnaire (see attachment 2 ) fitting our specific teaching context, we selected reasonable and appropriate items from a number of published instruments: Teacher charisma was assessed through a selection of items originating from four validated instruments [28], [29], [30], [31]. Didactic quality items were also derived from four pre-existing instruments [31], [32], [33], [34]. To measure digital affinity, items of a recent cluster analysis were used [26]. The wording of some items was mildly adapted to fit the seminar setting.

### 2.4. Statistics

RStudio 1.2.1335 (Boston, USA) and Microsoft PowerPoint (Redmond, USA) were used to create the illustrations. IBM SPSS Statistics 26.0 (Armonk, USA) and Mplus 7.11 (Muthén & Muthén, Los Angeles, USA) were used for statistical analyses. Missing information was treated statistically as absent, i.e. an imputation procedure was not conducted. Means and standard deviations were calculated for descriptive analysis. Cronbach’s alpha served as a measure of internal consistency. An acceptable alpha-value was defined as greater than 0.70 [35]. To ensure equality between the semesters and the seminar tests, the demographic data of students and the difficulty of the test questions were compared using Student’s t-test. The Welch test was employed using the teaching format as independent variable, and estimated learning gain, academic performance, teacher charisma, rating of didactic quality, and overall satisfaction as dependent variables to test for significance between groups [36]. Pearson correlation coefficients for each format were computed and tested for significance (p<0.05). As Pearson correlation of composite scores does not allow for the correction of measurement error, structural regression models of teacher charisma, digital affinity, and didactic quality were computed [37]. From a statistical point of view, results of structural regression models are more robust than simple Pearson correlations [38]. We set our hypothesis that didactic quality and teacher charisma correlated positively in all computed models. In model 1, the relationships derived from digital affinity were treated as regressions with digital affinity predicting both didactic quality and teacher charisma. Theory-compliant significant intercorrelations based on modification indices greater than 10 were not allowed in model 1, but allowed in an otherwise equivalent model 2. In contrast, all relationships were treated as correlations in model 3, in which intercorrelations based on modification indices greater than 10 were also allowed. The root mean square error of approximation (RMSEA), standardized root mean square residual (SRMR), and comparative fit index (CFI) were employed to investigate the model fit, in order to determine the final model. RMSEA determined how well the model with unknown but optimally chosen parameters would fit the population’s covariance matrix [39]. Its strength lies in its sensitivity to the number of estimated parameters in the model; values lower than 0.06 suggest a relatively good fit between the hypothesized model and the observed data [40]. SRMR serves as a standardized measure assessing the residuals of the sample covariance matrix and the hypothesised covariance model; values below 0.08 indicate a relatively good fit [40]. CFI constitutes an incremental fit index derived from the normed fit index, considering sample size. Values greater than 0.90 are recognized as indicative of a satisfactory fit. Where possible, simple linear regression analysis was performed to determine effect priority. Regression lines best fitting the data were plotted and described by the prediction formula Y=a+b*X.

## 3. Results

### 3.1. Descriptive statistics

There were 163, 143, and 147 students enrolled in the seminars in the winter term of 2016/2017, summer term of 2019, and winter term of 2019/2020, respectively. The end-of-semester survey on didactic quality, overall satisfaction with the seminars, and student digital affinity was completed by 103, 125, and 97 students, respectively. In the winter term of 2019/2020, a total of 599 quick-response evaluations were collected, assessing estimated learning gain, academic performance, and teacher charisma (tablet-based n=422, presentation-based n=177) with 138 students having evaluated at least once. There were no significant differences with respect to the demographic data on comparison of the study participants in the three semesters (55.6% female, mean age 23.95±3.17 years, mean semester 6.97±0.22, p>0.05). Furthermore, no significant differences were found in the difficulty of the test questions used in tablet-based teaching compared to those used in presentation-based seminars (p>0.05). Cronbach’s alpha of the rated level of competence related to the learning objectives was α=0.84 (before the seminar) and α=0.72 (after completion of the seminar) and therefore considered good. Cronbach’s alpha of the teacher charisma and didactic quality scales was excellent (both α=0.87), whereas that of the digital affinity scale was only 0.62. Containing only four items, this value was just below the limit of being acceptable. Comparing all scales employed (mean teacher charisma 4.47±0.52, mean didactic quality 4.31±0.48, mean digital affinity 3.44±0.71, mean overall satisfaction 4.46±0.63), students varied mostly in their digital affinity, which they rated as rather low.

### 3.2. Scores of estimated learning gain, academic performance, teacher charisma, didactic quality, and overall satisfaction

**Figure 1:**
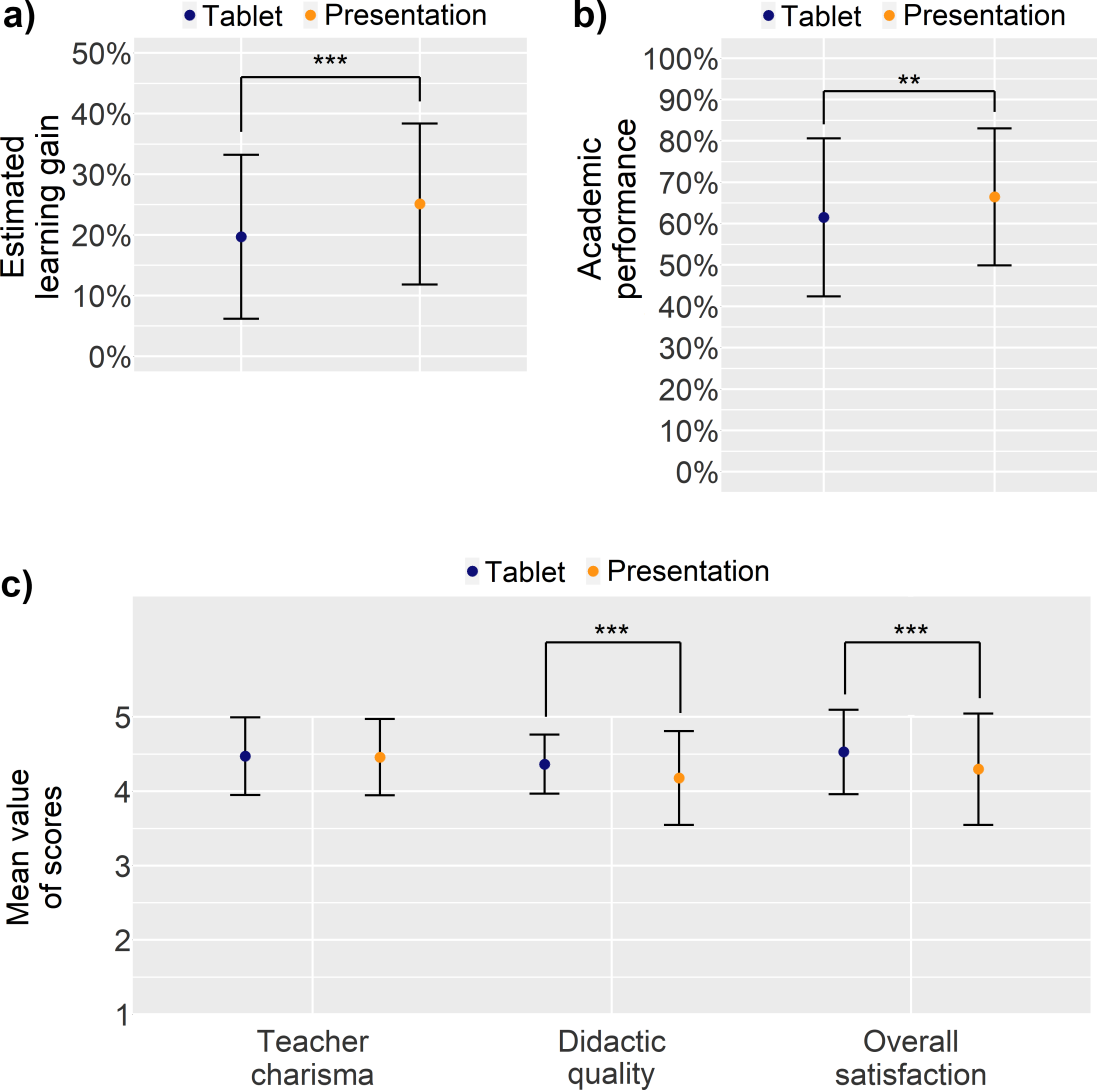
Plots of means and error bars of estimated learning gain (a), academic performance (b) and teacher charisma, didactic quality, and student’s overall satisfaction (c) for the tablet-based and the presentation-based seminars. Error bars indicate ± standard deviation. Horizontal bars indicate significance between groups and effect size; **p<0.01, ***p<0.001. As student digital affinity was assessed independently of the seminar format, it is not depicted in this figure. Please note that estimated learning gain, teacher charisma, and academic performance were only assessed in the winter term of 2019/20.

Presentation-based seminars yielded significantly higher scores in the estimated learning gain as well as in the test of academic performance (see figure 1 a and b (Fig. 1)). However, the tablet-based teaching format surpassed the traditional, presentation-based format significantly in the students’ evaluation of didactic quality and overall satisfaction (see figure 1 c (Fig. 1)). Of note, both teaching formats scored well. No differences were found in the rating of teacher charisma with respect to each teaching format. 

### 3.3. Impact of teacher charisma and student digital affinity in tablet-based seminars

All of the following analyses were conducted using only the data from the last semester of the study (winter term of 2019/20). Pearson correlation coefficients of all explored variables were computed for tablet-based and presentation-based seminars (see table 2 (Tab. 2) and table 3 (Tab. 3)). In both teaching formats, estimated learning gain and academic performance did not correlate significantly. However, in the tablet-based seminar alone, a significant correlation existed between estimated learning gain and teacher charisma, indicating a greater impact of teacher charisma in a technology-based format. In both formats, overall satisfaction with the seminar highly correlated with evaluation of the didactic quality. Additionally, in the tablet-based seminar, student digital affinity correlated significantly with overall satisfaction and didactic quality. As these correlations were not significant in the traditional seminar format, digital affinity seems to be particularly relevant in student perception of the tablet-based seminar.

### 3.4. Structural regression model of digital affinity, teacher charisma, and didactic quality

Comparing the three structural regression models computed, model 2 (hypothesizing a regression of digital affinity predicting didactic quality and allowing intercorrelations) yielded the best goodness-of-fit indices for modelling the tablet-based seminars (a) as well as the presentation-based seminars (b): RMSEA=0.05(a), 0.05(b); SRMR=0.07(a), 0.07(b); CFI=0.90(a), 0.92(b). All further analyses were therefore based on model 2. 

**Figure 2:**
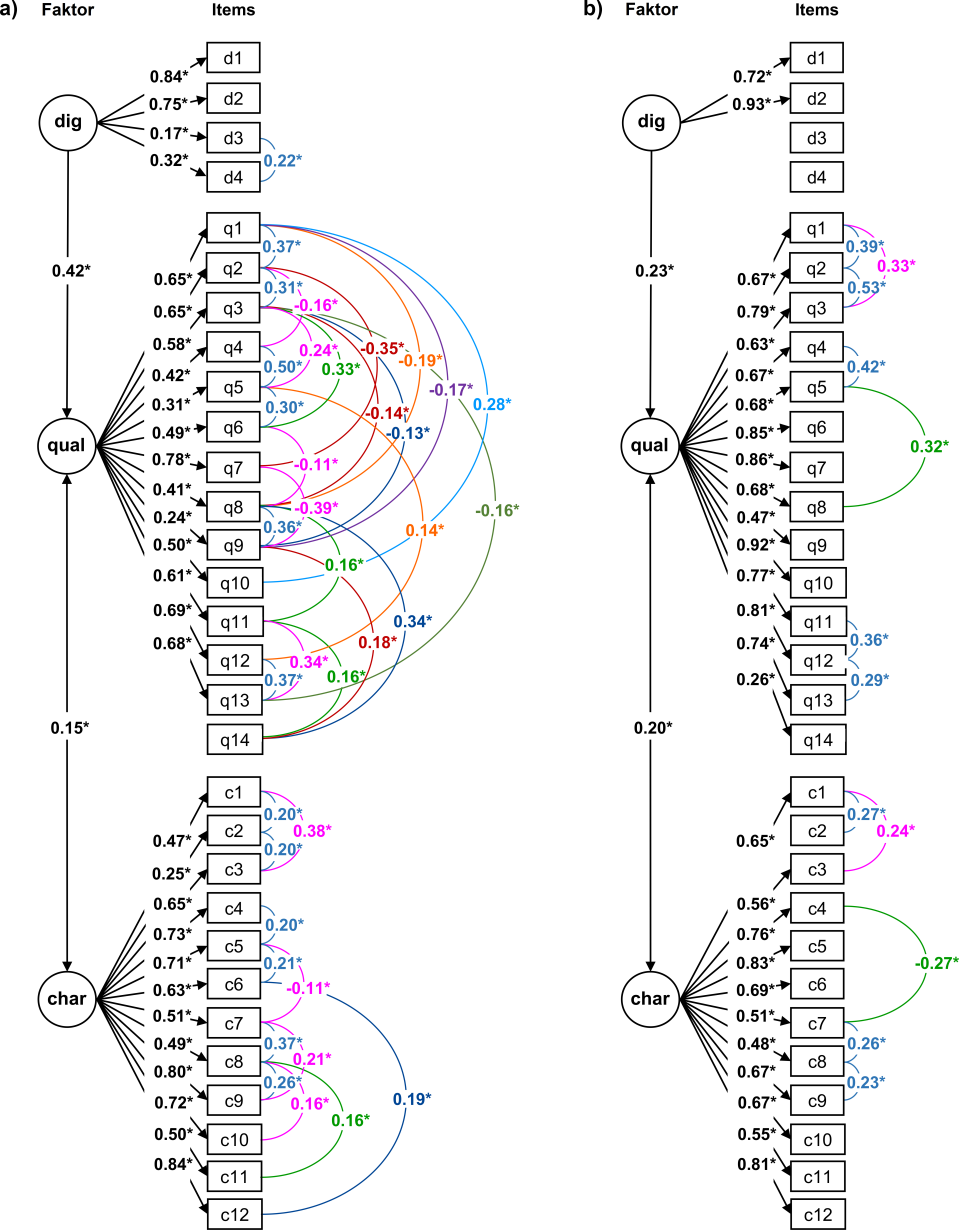
Structural regression model depicting the relationships between digital affinity (dig), didactic quality (qual), and teacher charisma (char) in tablet-based (a) and presentation-based seminars (b). Only significant coefficients are depicted. Single-headed arrow=regression, double-headed arrow=correlation, arch=intercorrelation, *p<0.05

The regression of digital affinity predicting didactic quality was significant in the models of both teaching formats (see figure 2 (Fig. 2)). The coefficient was more pronounced in tablet-based compared to presentation-based seminars (0.42 vs. 0.23). The correlation between teacher charisma and didactic quality was also significant with coefficients of similar size in both models (0.15 vs. 0.20). Students that perceived their teacher as charismatic rated the didactic quality higher, irrespective of the didactic format. The effect of digital affinity on teacher charisma was not significant.

Twelve intercorrelations were allowed in the modelling of presentation-based and 37 in the modelling of tablet-based seminars. Numerous intercorrelations originated from good organization (item q1), comprehensible learning objectives (item q3), and optimal variation of learning activities (item q8), but particularly in the model of tablet-based seminars. These items were linked to the oral participation of students in the seminar (item q9), acquisition of knowledge (item q11), understanding of radiology (item q13), and the desire to choose an imaging-based specialty for postgraduate training in the future (item q14). 

### 3.5. Moderating effect of teaching format on the relationship between digital affinity and didactic quality

**Figure 3:**
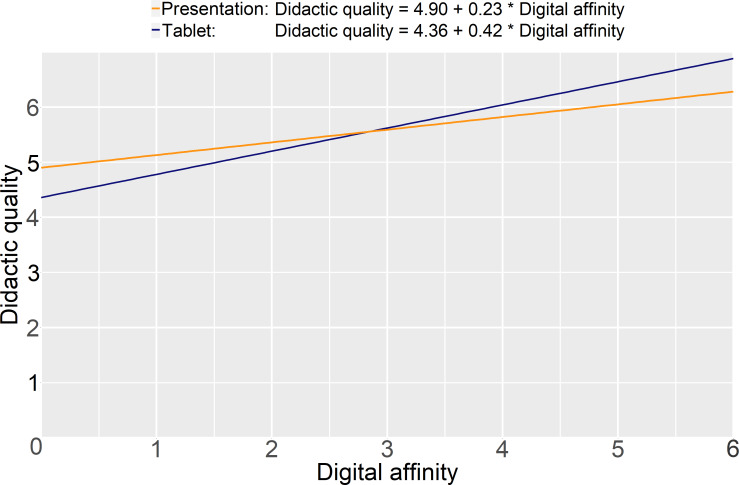
Regression analysis depicting the moderating effect of teaching format on the relationship between digital affinity and didactic quality. Please note that regression analysis estimates the hypothetical relationship, and theoretically, it is possible to plot values outside the response option range of 1-5, as illustrated.

The teaching format had a moderating effect on the relationship between digital affinity and didactic quality, as illustrated in figure 3 (Fig. 3). An increase in digital affinity would thus lead to a greater increase in the didactic quality of the tablet-based seminars compared to presentation-based seminars. The model suggests that the ratings of didactic quality for the two teaching formats diverge in the extremes of digital affinity: Low levels of digital affinity lead to a higher rating of didactic quality in the presentation-based seminars, whereas high levels of digital affinity lead to a higher rating of didactic quality in the tablet-based format. 

## 4. Discussion

While the implementation of tablet devices in medical education has been the topic of numerous studies, a direct comparison with a traditional teaching method has only seldom been performed [41], [42], [43]. In order to elaborate on the existing literature and to explore the impact of tablet-based teaching and its covariates, we conducted this study involving an undergraduate radiology seminar. 

In our study, we were unable to declare any one of the teaching formats as superior. Both the tablet-based and the presentation-based seminars yielded high ratings in didactic quality and overall satisfaction from students, however with a statistically significant difference in favour of the tablet-based seminars. Students perceived both didactic formats well, with a preference for the tablet-based format, despite its higher cognitive demand. Nevertheless, the presentation-based seminars proved superior in academic performance as well as in the estimated learning gain. We were able to demonstrate a complex interplay of factors affecting students’ experience when comparing the two teaching formats.

### 4.1. The role of teacher charisma in tablet-based seminars

With respect to the tablet-based seminars, our results indicate that teacher charisma positively correlates with the estimated learning gain and students’ rating of the didactic quality. Charismatic teachers are known to convey enthusiasm and rapport, thereby eliciting student interest and participation [44], [45]. Students may attribute this induced engagement to high didactic quality, which is also linked to teacher charisma. In the literature, a similar influence of teacher charisma on perceived teacher effectiveness has been demonstrated [17], [18]. The physical attractiveness of a teacher appears to modify this relationship [17], raising the question of other possible confounding factors. However, the use of digital media does not seem to be an obstacle to teacher personality [22]. Our findings suggest that charisma might play an even greater role in modern digitized teaching formats. Whilst teacher charisma correlated with ratings of didactic quality in both formats, it only had an impact on student estimated learning gain in the tablet-based seminar. This effect may be attributed to a desired shift towards learner-centred teaching, which is known to be facilitated by digital media [6], [46]. A highly charismatic teacher is also more likely to fulfil the appealing role of a moderator, who supports students in their self-directed, e.g. tablet-based learning process and therefore stimulates the perception of an enhanced learning outcome. In fact, studies indicate that charisma can be trained and may lead to improved student ratings in teaching [47], [48]. A reflection of teacher personality should therefore be conducted, particularly when introducing digital media into teaching concepts.

### 4.2. The role of digital affinity of students in tablet-based seminars

Our results also suggest that the digital affinity of students plays a major role in the tablet-based seminars, since it correlated with their overall satisfaction and rating of didactic quality. We were also able to document a moderating effect of the teaching format: The effect of higher digital affinity leading to higher ratings of didactic quality was especially pronounced in the tablet-based format. This is consistent with the finding that students with positive attitudes towards computers deem courses to be more satisfactory and efficient [49]. They tend to choose digital learning formats as their preferred learning environment to profit the most [50]. Another study was also able to reveal a significant relationship between online self-efficacy and three factors concerning interactivity with user satisfaction and perceived learning in an online course [51]. Among all factors, online self-efficacy was found to be the most significant predictor of perceived learning, which also accords well with our results. 

In our study, undergraduate medical students with high digital affinity showed a preference for practicing the interpretation of imaging results in tablet-based seminars that enabled them to access images through PACS. Yet students varied to the greatest extent in their self-rating of digital affinity, the rating of which was the lowest on average of all variables. This underlines the importance of not taking a high degree of digital affinity in students for granted. Regression analysis revealed that, on the spectrum of digital affinity, high levels may further improve the learning experience with digital media and low levels might even hinder the process. An assessment of digital affinity and compensatory support for users lacking the necessary skills should therefore be conducted prior to the implementation of tablet-based learning wherever possible [52].

### 4.3. Alignment of objectives, effective teaching methods, and assessment of students

In spite of the wide acceptance of the tablet-based teaching concepts in our study, estimated learning gain and academic performance by students did not reach the corresponding levels set in traditional face-to-face teaching. While this finding appears somewhat contrary to one of the reasons for introducing tablet devices, it sheds light on a potential pitfall of teaching with tablet devices and provides guidance on how to avoid it: The introduction of digital devices into pre-existing teaching programmes calls for an implementation strategy. Creating an educational concept of an aligned curriculum based on an intended learning outcome, planned teaching, as well as learning activities and assessment thereof, is a decisive task to ensure high levels of achievement in students. In our study, a lack of alignment may have provoked the discrepancy in estimated learning gain and academic performance between the two teaching formats. While the knowledge and image interpretation tests primarily assessed declarative knowledge, the tablet-based seminar was not primarily designed to fulfil this objective. In fact, it was particularly focused on acquiring procedural knowledge through individual navigation employing the image analysis programme [53]. This included scrolling through numerous images and identifying the relevant areas as well as anatomical landmarks that help to determine the underlying features of a given disease. Actually, students may have rated and performed worse for two reasons: learning the practical skills required shortened the time available for acquiring factual knowledge, and practical skills were not assessed in the academic performance test. These findings highlight the described necessity to design a seminar carefully; pointing out that the implementation of a new, authentic way of teaching needs curricular alignment, which includes the correct assessment method. Advantages and disadvantages of such new teaching methods need to be evaluated: Giving students authentic clinical tasks, as conducted in the tablet-based seminars, can contribute to improving communication, collaboration and problem-solving skills as well as increase student confidence [54]. The close correspondence between the educational and the expected future workspace tasks can also lead to increased student engagement, efforts and satisfaction [54]. Yet, the unfamiliarity and uncertainty about the demands of teaching formats of high task-authenticity can cause anxiety and stress for students [55] and the implementation can be time-consuming and costly [56]. Authentic educational methods may require extensive educational resources in order to achieve intended outcomes [57]. 

### 4.4. Didactic factors influencing the tablet-based seminar

The structural regression model in our study suggested that good organisation, comprehensible learning objectives, and an optimal variation of learning activities are crucial elements in the tablet-based seminars. In previous literature, these characteristics were also identified as important factors in digitally assisted formats. It has been demonstrated that mobile devices can promote learner-centred teaching and enhance the learner experience [5], [46]. Clear learning objectives allow students to gauge their understanding of the material, to self-direct their learning process towards the fundamental points, and to promote their learning outcome directly [58], [59]. However, it must be pointed out that teaching with mobile devices should not be conducted exclusively, but as one effective element in the variety of current and popular teaching methods [60].

### 4.5. Strengths of the study

The strength of our study lies in the fact that the same teachers delivered both the presentation-based and the tablet-based seminars to the same group of students. As a result, our findings comparing both formats should be attributed primarily to the differences in teaching format rather than teachers or student cohorts. Of note, we calculated the value of teacher charisma as perceived by each individual student and did not focus on the teacher’s mean charisma value across students, as the perception of charisma may vary greatly between individuals [61], [62].

### 4.6. Limitations and further research

There are some clear limitations associated with this study, which may serve as a starting point for further research. The study took place within the framework of the regular compulsory teaching in radiology, in which the academic progress of large numbers of students had to be ensured. We were therefore unable to vary the conditions for different study designs or cohorts, as we adopted the authentic environment of teaching and not some artificial classroom setup. We compared and contrasted the current standard of traditional presentation-based seminars as control with the new best practice of tablets and the PACS application. Thus, all students were subjected to exactly the same curriculum to guarantee equality.

It is also important to note that the data on estimated learning gain, academic performance, and teacher charisma were collected during only one of the three study semesters, which limits the generalizability of the findings regarding these variables. Nevertheless, a large number of 599 evaluations were included in the study, which were carried out by a total of 138 different students from one semester through multiple evaluations. 

Since our teaching intervention was domain-specific and tailored to the tablet-based seminar in the field of radiology, particular attention should be paid to whether the results can be generalized and applied to other subjects and specialties. Additionally, in order to do justice to a potential change in student digital affinity over time, systematic longitudinal studies on tablet use in medical teaching will be necessary.

Although the participating student groups did not vary significantly in gender, age, or semester, we cannot exclude confounders such as differences in socioeconomic and educational background, as well as prior medical experience. However, this well reflects the genuine heterogeneity of students, which represents one of the challenges of teaching in higher education. 

While the estimated learning gain was assessed using a retrospective post-then-pre design, academic performance was only assessed once after the teaching intervention, which removes the opportunity to control for pre-test bias. However, student groups attended the seminars in different orders; this effect on learning was therefore minimized. Nevertheless, where feasible, pre-test variability should be taken into account by using retrospective post-then-pre or classic pre-post approaches in further studies.

In future, we may better investigate different teaching formats with respect to identical content instead of different seminar subjects. Focus may also be placed on comparing teaching formats of equally high task-authenticity and comparable cognitive demands. Another avenue of future work is to identify potential confounding factors regarding the influence of teacher charisma such as physical attractiveness, gender, or language capabilities. 

## 5. Conclusion

In summary, this empirical study addresses gaps within the literature while highlighting learning delivery and factors influencing the perceived quality in tablet-based seminars. It becomes clear that the implementation of tablets alone is not sufficient to guarantee high-quality modern teaching. It is crucial to ensure that various factors concerning teachers, students, and didactics are in accordance with the needs, methods, and intended learning outcomes of teaching in the current digital age. As this study reinforces: *“It is not the technology but the instructional implementation of the technology that determines the effects on learning”* [63]. The findings of this study have a number of practical implications: New media and technical infrastructure appeal to the needs of state-of-the-art medical education. At the same time, motivated and inspiring teachers have to set a situation in which students can and will learn effectively whilst appreciating and/or enjoying the didactic quality. Allowing students to choose between different teaching formats based on their individual preferences and affinity, and aligning authentic teaching and learning activities with appropriate methods of assessment are just a few approaches to achieving this goal.

## Statements and declarations

### Ethics approval

The local institutional review and ethics board judged the project as not representing medical or epidemiological research on human subjects and as such adopted a simplified assessment protocol. The project was approved without any reservation under the proposal number 20210118 04.

### Consent to participate

All students and teachers provided written informed consent after receiving information on the study conditions.

## Data

Data for this article are available from Dryad Repository: [https://doi.org/10.5061/dryad.9p8cz8wm2] [64]

## Authors’ contributions

- Sandra Weigel: Conceptualization, formal analysis, investigation, methodology, visualization, writing (original draft), data curation
- Joy Backhaus: Conceptualization, formal analysis, data curation, software, writing (review and editing)
- Jan-Peter Grunz: Conceptualization, writing (review and editing)
- Andreas Steven Kunz: Conceptualization, writing (review and editing)
- Thorsten Alexander Bley: Supervision
- Sarah König: Conceptualization, project administration, supervision, visualization, writing (review and editing)

## Acknowledgements

We would like to take this opportunity to thank all the students who participated, without whom this study would never have been possible. Furthermore, we would like to thank Andrew Entwistle for providing comments on the draft version and the assistance with proofreading the manuscript.

## Competing interests

The authors declare that they have no competing interests. 

## Supplementary Material

Learning objectives

Questionnaire

## Figures and Tables

**Table 1:**
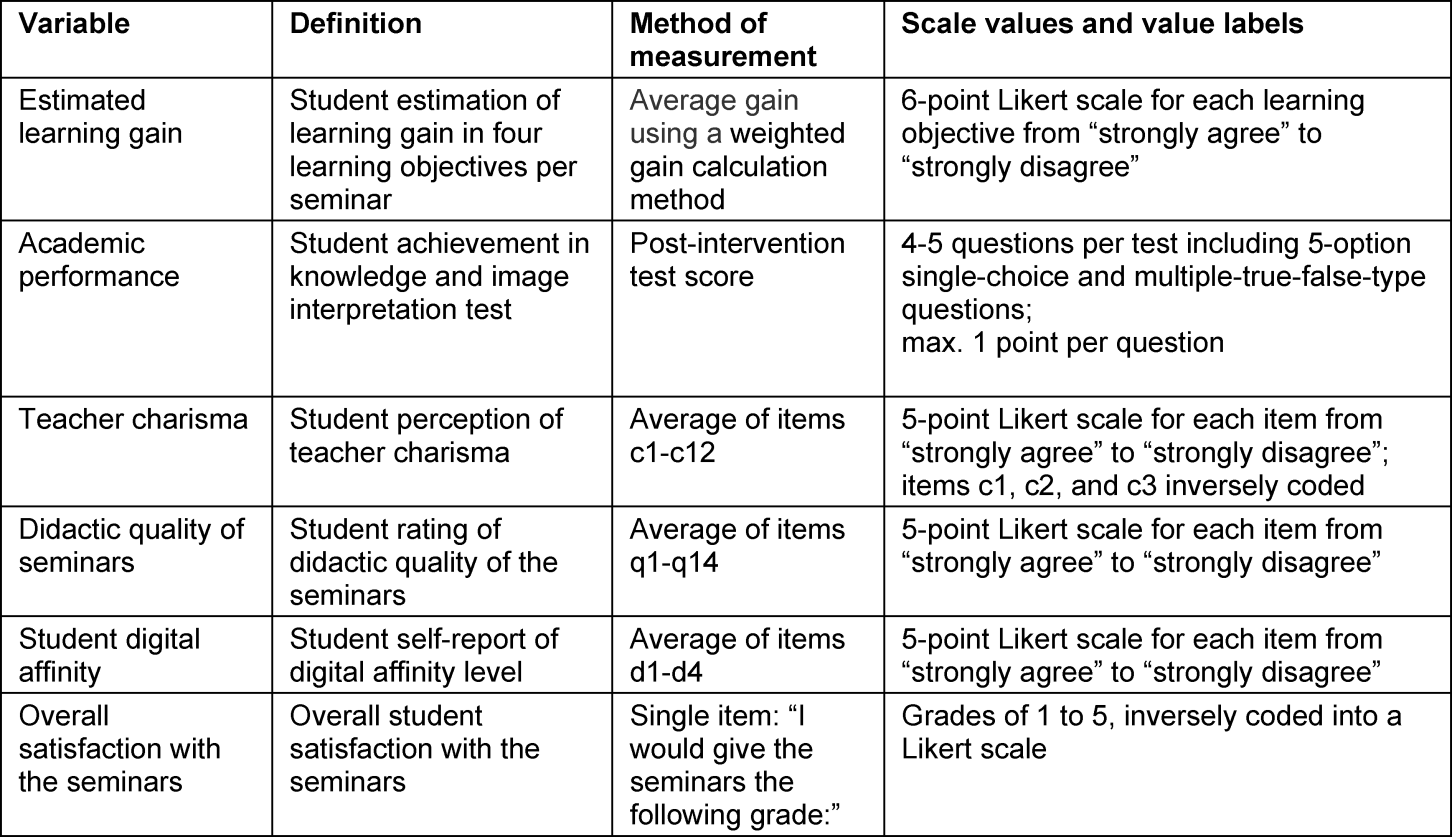
Variables studied

**Table 2:**
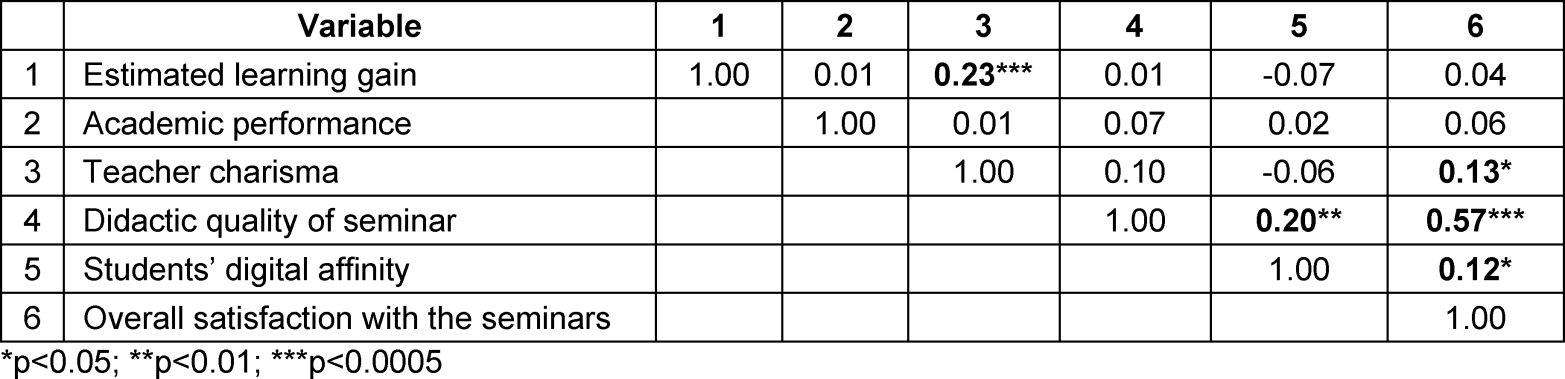
Pearson correlations between the variables in tablet-based seminars

**Table 3:**
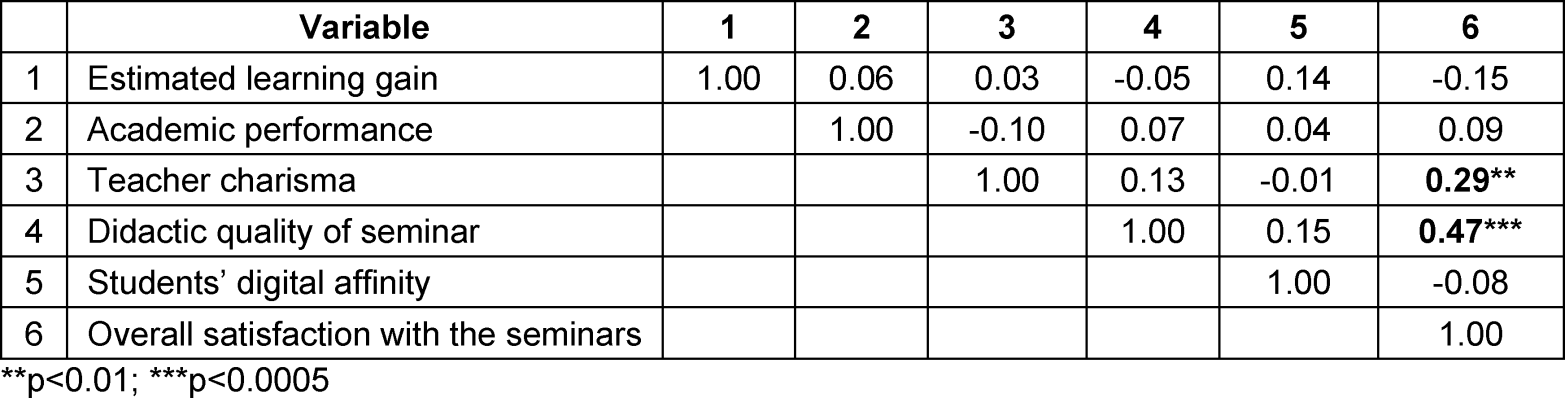
Pearson correlations between the variables in presentation-based seminars

## References

[1] Lu YC, Xiao Y, Sears A, Jacko JA (2005). A review and a framework of handheld computer adoption in healthcare. Int J Med Inform.

[2] Mickan S, Tilson JK, Atherton H, Roberts NW, Heneghan C (2013). Evidence of effectiveness of health care professionals using handheld computers: a scoping review of systematic reviews. J Med Internet Res.

[3] Fleischmann R, Duhm J, Hupperts H, Brandt SA (2015). Tablet computers with mobile electronic medical records enhance clinical routine and promote bedside time: a controlled prospective crossover study. J Neurol.

[4] Strayer SM, Semler MW, Kington ML, Tanabe KO (2010). Patient attitudes toward physician use of tablet computers in the exam room. Fam Med.

[5] Bedi HS, Yucel EK (2013). “I Just bought my residents iPads… now what?” The integration of mobile devices into radiology resident education. AJR Am J Roentgenol.

[6] Wilkinson K, Barter P (2016). Do mobile learning devices enhance learning in higher education anatomy classrooms?. J Pedagogic Develop.

[7] Zafar S, Safdar S, Zafar AN (2014). Evaluation of use of e-learning in undergraduate radiology education: a review. Eur J Radiol.

[8] Jeffrey D, Goddard P, Callaway M, Greenwood R (2003). Chest radiograph interpretation by medical students. Clin Radiol.

[9] Ochsmann EB, Zier U, Drexler H, Schmid K (2011). Well prepared for work? Junior doctors' self-assessment after medical education. BMC Med Educ.

[10] Silveira HD, Gomes MJ, Silveira HE, Dalla-Bona RR (2009). Evaluation of the radiographic cephalometry learning process by a learning virtual object. Am J Orthod Dentofacial Orthop.

[11] Kavadella A, Tsiklakis K, Vougiouklakis G, Lionarakis A (2012). Evaluation of a blended learning course for teaching oral radiology to undergraduate dental students. Eur J Dent Educ.

[12] Pusic MV, LeBlanc VR, Miller SZ (2007). Linear versus web-style layout of computer tutorials for medical student learning of radiograph interpretation. Acad Radiol.

[13] Vandeweerd JM, Davies JC, Pinchbeck GL, Cotton JC (2007). Teaching veterinary radiography by e-learning versus structured tutorial: a randomized, single-blinded controlled trial. J Vet Med Educ.

[14] Gibson KA, Boyle P, Black DA, Cunningham M, Grimm MC, McNeil HP (2008). Enhancing evaluation in an undergraduate medical education program. Acad Med.

[15] Lee DC, Lu JJ, Mao KM, Ling SH, Yeh MC, Hsieh Cl (2014). Does teachers charisma can really induce students learning interest?. Procedia Soc Behav Sci.

[16] Lin S, Huang Y (2014). Examining teaching charisma and its relation to student engagement. Cross Cult Comm.

[17] Rannelli L, Coderre S, Paget M, Woloschuk W, Wright B, McLaughlin K (2014). How do medical students form impressions of the effectiveness of classroom teachers?. Med Educ.

[18] Shevlin M, Banyard P, Davies M, Griffiths M (2000). The validity of student evaluation of teaching in higher education: love me, love my lectures?. Assess Eval High Educ.

[19] Carini RM, Kuh GD, Klein SP (2006). Student engagement and student learning: Testing the linkages. Res High Educ.

[20] Kuh GD (2009). What student affairs professionals need to know about student engagement. J Coll Stud Develop.

[21] Ottenbreit-Leftwich AT, Glazewski KD, Newby TJ, Ertmer PA (2010). Teacher value beliefs associated with using technology: Addressing professional and student needs. Comput Educ.

[22] Towler A, Arman G, Quesnell T, Hoffman L (2014). How charismatic trainers inspire others to learn through positive affectivity. Comp Human Behav.

[23] Margaryan A, Littlejohn A, Vojt G (2011). Are digital natives a myth or reality? University students’ use of digital technologies. Comput Educ.

[24] Waycott J, Bennett S, Kennedy G, Dalgarno B, Gray K (2010). Digital divides? Student and staff perceptions of information and communication technologies. Comput Educ.

[25] Howard SK, Ma J, Yang J (2016). Student rules: Exploring patterns of students’ computer-efficacy and engagement with digital technologies in learning. Comput Educ.

[26] Backhaus J, Huth K, Entwistle A, Homayounfar K, Koenig S (2019). Digital affinity in medical students influences learning outcome: a cluster analytical design comparing Vodcast with traditional lecture. J Surg Educ.

[27] Westphale S, Backhaus J, Koenig S (2022). Quantifying teaching quality in medical education: The impact of learning gain calculation. Med Educ.

[28] Bolkan S, Goodboy AK (2014). Communicating charisma in instructional settings: Indicators and effects of charismatic teaching. Coll Teach.

[29] Huang YC, Lin SH (2014). Assessment of charisma as a factor in effective teaching. J Educ Technol Soc.

[30] Hirschfeld G, Thielsch M (2009). Münsteraner Fragebogen zur Evaluation von Vorlesungen (MFE-V).

[31] Staufenbiel T (2000). Fragebogen zur Evaluation von universitären Lehrveranstaltungen durch Studierende und Lehrende. Diagnostica.

[32] Gollwitzer M, Schlotz W (2003). Das "Trierer Inventar zur Lehrveranstaltungsevaluation"(TRIL): Entwicklung und erste testtheoretische Erprobungen. Psychologiedidaktik und Evaluation IV.

[33] Gediga G, Von Kannen K, Schnieder F, Köhne S, Luck H, Schneider B (2000). KIEL: Ein Kommunikationsinstrument für die Evaluation von Lehrveranstaltungen.

[34] Braun E, Gusy B, Leidner B, Hannover B (2008). Das Berliner Evaluationsinstrument für selbsteingeschätzte, studentische Kompetenzen (BEvaKomp). Diagnostica.

[35] Nunnally JC (1994). Psychometric theory 3E.

[36] Rasch D, Kubinger KD, Moder K (2011). The two-sample t test: pre-testing its assumptions does not pay off. Stat Papers.

[37] Geiser C (2012). Data analysis with Mplus.

[38] Schumacker RE, Lomax RG (2004). A beginner's guide to structural equation modeling.

[39] Byrne BM (1998). Structural equation modeling with LISREL, PRELIS, and SIMPLIS: Basic concepts, applications, and programming.

[40] Hu Lt, Bentler PM (1999). Cutoff criteria for fit indexes in covariance structure analysis: Conventional criteria versus new alternatives. Struct Equ Modeling.

[41] Patel S, Burke-Gaffney A (2018). The value of mobile tablet computers (iPads) in the undergraduate medical curriculum. Adv Med Educ Pract.

[42] Hill J, Nuss M, Middendorf B, Cervero R, Gaines J, Bastiaens T, Marks G (2012). Using iPads to Enhance Teaching and Learning in Third-Year Medical Clerkships.

[43] George P, Dumenco L, Doyle R, Dollase R (2013). Incorporating iPads into a preclinical curriculum: A pilot study. Med Teach.

[44] Erdle S, Murray HG, Rushton JP (1985). Personality, classroom behavior, and student ratings of college teaching effectiveness: A path analysis. J Educ Psychol.

[45] Frey PW (1978). A two-dimensional analysis of student ratings of instruction. Res High Educ.

[46] Ellaway R, Masters K (2008). AMEE Guide 32: e-Learning in medical education Part 1: Learning, teaching and assessment. Med Teach.

[47] Murray HG, Lawrence C (1980). Speech and drama training for lectures as a means of improving university teaching. Res High Educ.

[48] Towler AJ (2003). Effects of charismatic influence training on attitudes, behavior, and performance. Pers Psychol.

[49] Park JH, Wentling T (2007). Factors associated with transfer of training in workplace e‐learning. J Workplace Learn.

[50] Garcia F (2015). Using learner profiling technique to predict college students' tendency to choose elearning. Courses: a two-step cluster analysis. HETS Online J.

[51] Alqurashi E (2019). Predicting student satisfaction and perceived learning within online learning environments. Distance Educ.

[52] Murray MC, Pérez J (2014). Unraveling the digital literacy paradox: How higher education fails at the fourth literacy. Iss Inform Sci Inform Technol.

[53] Anderson J (1976). Language, memory, and thought.

[54] Sokhanvar Z, Salehi K, Sokhanvar F (2021). Advantages of authentic assessment for improving the learning experience and employability skills of higher education students: A systematic literature review. Stud Educ Eval.

[55] Forsyth H, Evans J (2019). Authentic assessment for a more inclusive history. High Educ Res Develop.

[56] Svinicki MD (2004). Authentic assessment: Testing in reality. New Dir Teach Learn.

[57] Jopp R (2020). A case study of a technology enhanced learning initiative that supports authentic assessment. Teach High Educ.

[58] Webb EM, Naeger DM, Fulton TB, Straus CM (2013). Learning objectives in radiology education: why you need them and how to write them. Acad Radiol.

[59] Sarrab M, Al-Shihi H, Al-Manthari B, Bourdoucen H (2018). Toward educational requirements model for Mobile learning development and adoption in higher education. TechTrends.

[60] Muyinda PB, Mugisa E, Lynch K (2007). M-learning: the educational use of mobile communication devices. Strengthening Role ICT Develop.

[61] Rosenberg A, Hirschberg J (2009). Charisma perception from text and speech. Speech Comm.

[62] Derrico F, Signorello R, Demolin D, Poggi I (2013). The Perception of Charisma from Voice: A Cross-Cultural Study.

[63] Collis B (1991). Anticipating the impact of multimedia in education: Lessons from literature. Int J Comput Adult Educ Train.

[64] Weigel S, Backhaus J, Grunz JP, Kunz AS, Bley TA, König S (2023). Data from: Tablet-based versus presentation-based seminars in radiology: effects of student digital affinity and teacher charisma on didactic quality.

