# Supplementary material for: Tablet-based versus presentation-based seminars in radiology: Effects of student digital affinity and teacher charisma on didactic quality
Source: GMS J Med Educ. 2023 Sep 15;40(5):Doc59. doi: 10.3205/zma001641 (PMC10594033; doi:10.3205/zma001641)
Supplement: Learning objectives [file JME-40-59-s-001.pdf]

Attachment 1: *Learning objectives*

**Gynaecological imaging**
I can explain the technical implementation of conventional mammography.
I can list the differential diagnoses of intramammary focal findings.
I can apply the BIRADS and ACR classifications.
I can evaluate alternative examination methods to mammography regarding their diagnostic strengths and weaknesses.
**Paediatric imaging**
I can explain the radiological peculiarities of paediatric images compared to radiological images of adults.
I can explain what typical paediatric pathologies look like in radiological imaging.
I can distinguish between "normal findings" and "abnormal findings" on paediatric images.
I can explain the ALARA principles.
**Imaging of the abdomen**
I can identify the anatomical structures of the abdomen on radiological images (including CT scans).
I can explain what typical abdominal pathologies look like in radiological imaging.
I can assess the strengths and weaknesses of different imaging modalities.
I can determine whether a radiological image of the abdomen represents a life-threatening acute pathology.
**Imaging of the heart and large vessels**
I can identify the anatomical structures of the heart on radiological images.
I can explain what typical pathologies of the heart and major vessels look like in radiological imaging.
I can explain the advantages and disadvantages of cardio-CT and cardio-MRI.
I can explain the technical basics of cardio-CT.
**Imaging of the thorax**
I can identify the anatomical structures in the chest X-ray.
I can explain what typical pathologies look like on chest X-ray.
I can independently evaluate a chest X-ray.
I can explain the differences between a.p. and p.a. projections.
**Imaging of the musculoskeletal system**
I can identify the different spatial planes in sectional imaging (axial/sagittal/coronal).
I can explain what typical acute bone pathologies look like in radiological imaging.
I can distinguish between “normal findings” and “abnormal findings” on images of the musculoskeletal system.
I can assess the need for surgical treatment based on diagnostic imaging.

Attachment 1 to Weigel S, Backhaus J, Grunz JP, Kunz AS, Bley TA, König S. *Tablet-based versus presentation-based seminars in radiology: effects of student digital affinity and teacher charisma on didactic quality*. GMS J Med Educ. 2023;40(5):Doc59. DOI: 10.3205/zma001641
