# Supplementary material for: Tablet-based versus presentation-based seminars in radiology: Effects of student digital affinity and teacher charisma on didactic quality
Source: GMS J Med Educ. 2023 Sep 15;40(5):Doc59. doi: 10.3205/zma001641 (PMC10594033; doi:10.3205/zma001641)
Supplement: Questionnaire [file JME-40-59-s-002.pdf]

Attachment 2: Questionnaire

c1	The teacher spoke in a dull voice.
c2	The teacher avoided eye contact with the students.
c3	The teacher had a blunt face.
c4	The teacher integrated gestures congruent to his words.
c5	The teacher had a lively way of speaking.
c6	The teacher communicated in a humorous fashion.
c7	The teacher was open to the concerns of the students.
c8	The teacher responded adequately to the students' questions and suggestions.
c9	The teacher ensured a positive working climate.
c10	The teacher showed interest in my learning progress.
c11	The teacher is an expert in his field.
c12	I could imagine the teacher as a mentor.
q1	The seminars are well organized.
q2	The seminars follow a clear structure.
q3	The learning objectives are clearly defined and comprehensible.
q4	In the seminars, there is an open climate for personal contributions.
q5	Following and thinking through the material/topic is encouraged.
q6	A connection between the basics of imaging and its use in clinical questions is established.
q7	The image materials used helped me a lot to work out the material.
q8	The variation of the learning activities (e.g. image analysis, interaction with fellow students, discussion etc.) is optimal for me.
q9	I contributed orally to the seminars.
q10	Digital media/images are integrated into the seminars sensibly.
q11	My knowledge is considerably greater after the seminars than before.
q12	I have learned something useful and important for medical practice.
q13	My understanding of radiology has developed through the seminars.
q14	My desire has grown to choose an imaging subject for my later continuing education.
d1	I am technically interested.
d2	I am good at using smartphones.
d3	Social networks play an important role for me.
d4	I enjoy reading in digital format.

Attachment 2 to Weigel S, Backhaus J, Grunz JP, Kunz AS, Bley TA, König S. *Tablet-based versus presentation-based seminars in radiology: effects of student digital affinity and teacher charisma on didactic quality*. GMS J Med Educ. 2023;40(5):Doc59. DOI: 10.3205/zma001641
